# Sensitivity to inhibition of DNA repair by Olaparib in novel oropharyngeal cancer cell lines infected with Human Papillomavirus

**DOI:** 10.1371/journal.pone.0207934

**Published:** 2018-12-13

**Authors:** Evelyne F. Pirotte, Stefan Holzhauser, David Owens, Stuart Quine, Ali Al-Hussaini, Adam D. Christian, Peter J. Giles, Stephen T. Man, Mererid Evans, Ned G. Powell

**Affiliations:** 1 HPV Research Group, Cardiff University School of Medicine, Cardiff, United Kingdom; 2 Ear Nose Throat / Head and Neck Surgery Department, University Hospital of Wales, Cardiff, United Kingdom and Vale University Health Board, Cardiff, United Kingdom; 3 Department of Pathology, University Hospital of Wales, Cardiff, United Kingdom and Vale University Health Board, Cardiff, United Kingdom; 4 Wales Gene Park, Cardiff University, Cardiff, United Kingdom; 5 Division of Cancer and Genetics, Cardiff University School of Medicine, Cardiff, United Kingdom; 6 Velindre Cancer Centre, Cardiff, United Kingdom; University of South Alabama Mitchell Cancer Institute, UNITED STATES

## Abstract

The incidence of Human Papillomavirus (HPV)-associated oropharyngeal squamous cell carcinoma (OPSCC) is increasing rapidly in the UK. Patients with HPV-positive OPSCC generally show superior clinical responses relative to HPV-negative patients. We hypothesised that these superior responses could be associated with defective repair of DNA double strand breaks (DSB). The study aimed to determine whether defective DNA repair could be associated with sensitivity to inhibition of DNA repair using the PARP inhibitor Olaparib. Sensitivity to Olaparib, and induction and repair of DNA damage, were assessed in a panel of 8 OPSCC cell-lines, including 2 novel HPV-positive lines. Effects on cell cycle distribution and levels of PARP1 and p53 were quantified. RNA-sequencing was used to assess differences in activity of DNA repair pathways. Two HPV-positive OPSCC lines were sensitive to Olaparib at potentially therapeutic doses (0.1–0.5 μM). Two HPV-negative lines were sensitive at an intermediate dose. Four other lines, derived from HPV-positive and HPV-negative tumours, were resistant to PARP inhibition. Only one cell-line, UPCISCC90, showed results consistent with the original hypothesis i.e. that in HPV-positive cells, treatment with Olaparib would cause accumulation of DSB, resulting in cell cycle arrest. There was no evidence that HPV-positive tumours exhibit defective repair of DSB. However, the data suggest that a subset of OPSCC may be susceptible to PARP-inhibitor based therapy.

## Background

Oropharyngeal Squamous Cell Carcinoma (OPSCC) develop in the tonsils, base of tongue, pharyngeal wall and soft palate. These tumours have historically been associated with tobacco and alcohol consumption, but in recent decades, many parts of the developed world have seen a rapid and dramatic rise in incidence of OPSCC caused by Human Papillomavirus (HPV) [1–3]. Multiple studies have demonstrated that patients with HPV-positive OPSCC survive significantly longer than patients with HPV-negative disease, despite commonly presenting with clinico-pathological features usually associated with poor prognosis (e.g. nodal metastases and extracapsular spread) [4–7]. The increased duration of survival in HPV-positive OPSCC means that patients may live for many years with the late side-effects of treatment, hence reducing the toxicity of treatments has become a priority. This need can partly be addressed through clinical trials which de-intensify treatment [8–10]. Reduced toxicity could also be achieved through greater use of targeted HPV-specific therapies. However, preclinical assessment of potential novel therapies is hampered by a lack of relevant *in vitro* models. HPV-positive OPSCC is a relatively new disease entity and only a few validated cell-lines derived from treatment-naïve HPV-positive OPSCC, typically tonsil cancers, are available worldwide [11]. There are hence parallel needs for additional cell-lines models of HPV-positive OPSCC, and for rationally targeted therapies to maintain good outcomes whilst reducing treatment-related late toxicities.

It has been proposed that the better prognosis of HPV-positive OPSCC patients, relative to HPV-negative patients, is partly attributable to greater intrinsic cellular radio-sensitivity [11,12]. It is well established that in productive infections, in which the viral life cycle is completed and new virus particles are produced, HPV subverts components of the DNA damage response, including ATM, ATR, FANCD2, BRCA1, to facilitate differentiation-dependent replication of its genome [13,14]. However, the relationship between the DNA damage response and HPV in the context of non-productive infections, associated with carcinogenesis, is less well defined. Several studies have suggested that expression of HPV oncogenes in tumour cells is associated with defects in repair of DNA double strand breaks (DSB), possibly through down regulation of DNA-PK and BRCA2 [15,16], potentially conferring a “BRCA-like” phenotype on HPV-infected cells. Given this possibility, we hypothesised that HPV-positive OPSCC might be vulnerable to synthetic lethal therapy using PARP inhibition. Our model proposed that inhibition of repair of DNA single strand breaks (SSB) would be followed by conversion of SSB to DSB during replication, and cells with less effective repair of DSB, i.e. a BRCA-like phenotype, would accumulate DSB, resulting in cell cycle arrest and possibly apoptosis. Hence the current study aimed to derive and characterise novel cell-lines from HPV-positive OPSCC, and use these and other validated cell-lines, to investigate the potential of a synthetic lethal approach to treatment of OPSCC. Sensitivity to the PARP inhibitor Olaparib and its effects on cell cycle and levels of DNA DSB, and p53 protein were assessed. This work was undertaken with the aim of generating preclinical data to support targeted treatments for HPV-positive disease.

## Materials and methods

### Patients, cell-lines and cell culture conditions

Primary oropharyngeal tumour biopsies were obtained from patients prior to treatment for OPSCC at Cardiff and Vale University Health Board, from February 2013 to May 2014. The study was conducted with NHS Research Ethics Committee approval: Research Ethics Committee for Wales 13/WA/0002. Written informed consent was obtained for use of all patient samples. The study was performed in accordance with the Declaration of Helsinki. Culture methods were based on those of Stanley *et al*. [17]; briefly, fresh biopsies transported immediately to the laboratory and coarsely minced then incubated with Glasgow Modified Eagles Medium with 10% Fetal Bovine Serum, 10ng/ml Epidermal Growth Factor, 500 ng/ml Hydrocortisone, 0.1 nM Cholera toxin, and 1x Penicillin/Streptomycin (Sigma-Aldrich, Gillingham, UK). Cultures were supplemented with 60 Gy irradiated 3T3 fibroblasts. All cell cultures were incubated at 37°C in 5% CO_2_. HPV status was assessed by p16 immunohistochemistry on the diagnostic biopsy, and HPV GP5/6 and E6 PCR on tumour DNA [5].

UMSCC4 (HPV-negative, tonsil SCC), UMSCC6 (HPV-negative, base of tongue SCC), UMSCC19 (HPV-negative, base of tongue SCC), UMSCC74a (HPV-negative, base of tongue SCC), UMSCC47 (HPV-positive, lateral tongue SCC) cell lines were all obtained from the laboratory of Professor Thomas Carey (University of Michigan, USA) [18,19]. UPCISCC90 (HPV-positive, base of tongue SCC) was obtained from DSMZ (Braunschweig, Germany). These lines were obtained in October 2013 and were grown in DMEM with 10% Fetal Bovine Serum. The identities of these lines were confirmed by analysis of Short Tandem Repeat (STR) loci.

Human Epithelial Keratinocytes (HEKn) were grown in EpiLife media supplemented with 1% Human Keratinocyte Growth Supplement (HGKS) (cells and media from Thermo Fisher Scientific, Waltham, USA).

### Nucleic extraction and analysis

DNA and RNA were extracted using QiaAMP DNA and RNA Mini Kits, according to the manufacturer’s instructions (Qiagen, Hilden, Germany). p53 status was determined by bi-directional sequencing of exons 2 to 11 and alignment with p53 genomic sequence NC_000017.9 [20]. STR analysis was performed using the ABI AmpFISTR Identifiler kit according to the manufacturer’s instructions (Life Technologies, Carlsbad, USA), followed by analysis on an ABI 3130xl Genetic Analyser (Thermo Fisher Scientific, Waltham, USA).

### mRNA sequencing

RNA transcriptome sequencing was performed at the Beijing Genomics Institute (TruSeq RNA-seq protocol using Illumina HiSeq2000 to generate 90 bp paired-end reads). Quality control was performed using FastQC (Babraham Bioinformatics: http://www.bioinformatics.babraham.ac.uk/projects/fastqc/). Initial mapping against human reference hg19 and HPV16 reference NC_001526.2 genome was performed using TopHat (version 2.0.8b) [21]. Gene expression level was determined using RPKM (Reads per Kilobase exon Model per million mapped reads) values for exons and transcripts using the RefSeq gene model RefGene for hg19 and NC_001526.2.

Differentially Expressed Genes (DEG) were identified using edgeR analysis [22] on normalized count data with the Benjamini–Hochberg FDR method to account for multiple testing and false discovery [23]. A p value of <0.05 was interpreted as statistically significant. A list of the top 500 DEGs were subjected to over-representation analysis against Gene Ontology annotations using the GOseq method with FDR correction [24]. Paired-end reads mapping to both hg19 and NC_001526.2 were used to identify HPV:human fusion transcripts and generate Circos plots [25].

### Clonogenic survival assays

Cells were plated at low density (2500–12500 cells/plate depending on cell line) in 6cm diameter TC dishes and incubated for 24 h. Media was then replaced with media containing Olabparib (AZD2281—Selleckchem, Munich, Germany) or vehicle alone; DMSO concentration was standardised for all cultures at 0.1%. After 7 days the media was replaced with fresh media without Olaparib. After a further 7 days, media was removed and the cells were stained with crystal violet. Colonies containing more than 50 cells were counted– 50 cells is a standard threshold that allows macroscopic counting of colonies [26]. All experiments were performed in triplicate and repeated 3 times.

### Cell cycle and γ-H2AX

Cell cycle distribution and levels of γ-H2AX (to quantify DNA DSB) were determined in prepared nuclei by flow cytometry using DAPI (Sigma-Aldrich, UK) and anti-γ-H2AX antibody (clone JBW301) fluorescein isothiocyanate (FITC) conjugate (Merck-Millipore, Watford, UK; catalogue 16-202A) using published methodology [27]. Briefly, nuclei were prepared in lysis buffer containing 320mM Sucrose, 10mM TRIS-HCl pH8, 2.5mM MgCl2 and 0.5% Triton X-100, then filtered through a 20 μm CellTrics filter (Sysmex, Milton Keynes, UK). Flow cytometry was performed using a BD FACSCanto II flow cytometer (BD Biosciences, New Jersey, USA) with excitation at 405 and 488nm. Data analysis was performed using FlowJo software (FlowJo LLC, Ashland, USA).

### Western blot analysis

Cells at 40% confluence had standard media replaced with media including varying concentrations of Olaparib. They were then incubated for a further 48hr. Protein lysates were then prepared according to standard protocols using reagents from Sigma-Aldrich, St Louis USA. 20μg of protein was separated by SDS-PAGE electrophoresis. Western blots were performed using the following antibodies: p53 (rabbit) cat-9282; phospho-p53 (Ser15) (rabbit) cat-9284; PARP1 antibody (rabbit) cat-9542; β-Actin (13E5) antibody (rabbit) cat-4970 –all from Cell Signalling, Danvers, USA; and goat polyclonal secondary to rabbit IgG adsorbed cat: ab97080 (Abcam, Cambridge UK). Bands were visualised using the ECL Prime Chemiluminescence system (GE Healthcare, Chicago USA). Image capture was performed using a LAS-3000 imager (Fujifilm) using an initial 30 s exposure then 2 min increments. Quantification was performed using Image Studio Lite software (LI-COR Biotechnology, Cambridge, UK).

## Results

### Derivation of novel HPV-positive OPC cell-lines

Explant culture was attempted using fresh primary tumour biopsies from 12 patients with suspected OPSCC. Eight biopsies produced explants but did not proliferate beyond a few passages; two biopsies did not produce explants. Two novel cell lines, CUOP2 and CUOP3, were generated from the two biopsies in which sustained proliferation was achieved. These lines have now been grown to approximately 100 and 80 population doublings respectively and appear to be immortal. CUOP2 was derived from a p16-positive (>70% diffuse staining) T4aN2bM0 tonsil tumour in a male, 52-year-old, never smoker. CUOP3 was derived from a p16-positive T1N2aM0 tonsil tumour in a male, 44-year-old, ex-smoker with less than 10 pack/year history (Table 1). DNA and mRNA sequence data indicated the presence of HPV16 and wild-type p53 in both lines. DNA fingerprinting, using Short Tandem Repeat analysis, confirmed that the cell-lines were derived from the original patient biopsies. Both lines tested negative for mycoplasma and expression of p16 was confirmed by immunohistochemistry (S1 Fig).

**Table 1:** CUOP2 and CUOP3 patient characteristics.

Study number	Sex	Age (yrs)	Smoking status^a^	Site	TNM Stage	p16 IHC	P53 status	HPV type	Treatment^b^	Recurrence^c^
CUOP2	M	52	Never smoker	Left tonsil	T4aN2bM0	Positive	Wt	16	ChemoRT	None
CUOP3	M	44	Ex-smoker, <10pack/yr	Right tonsil	T1N2aM0	Positive	Wt	16	Transoral laser resection, neck dissection, post-op RT,	None

^a^ Pack-years refers to the number of cigarette packs smoked per day multiplied by the number of years the person has smoked.

^b^ Radiotherapy (RT), post-operative (post-op).

^c^ Recurrence as reported in September 2017.

### Transcriptomic analysis indicates similar DNA repair activity in HPV-positive and negative cell lines

**Fig 1:**
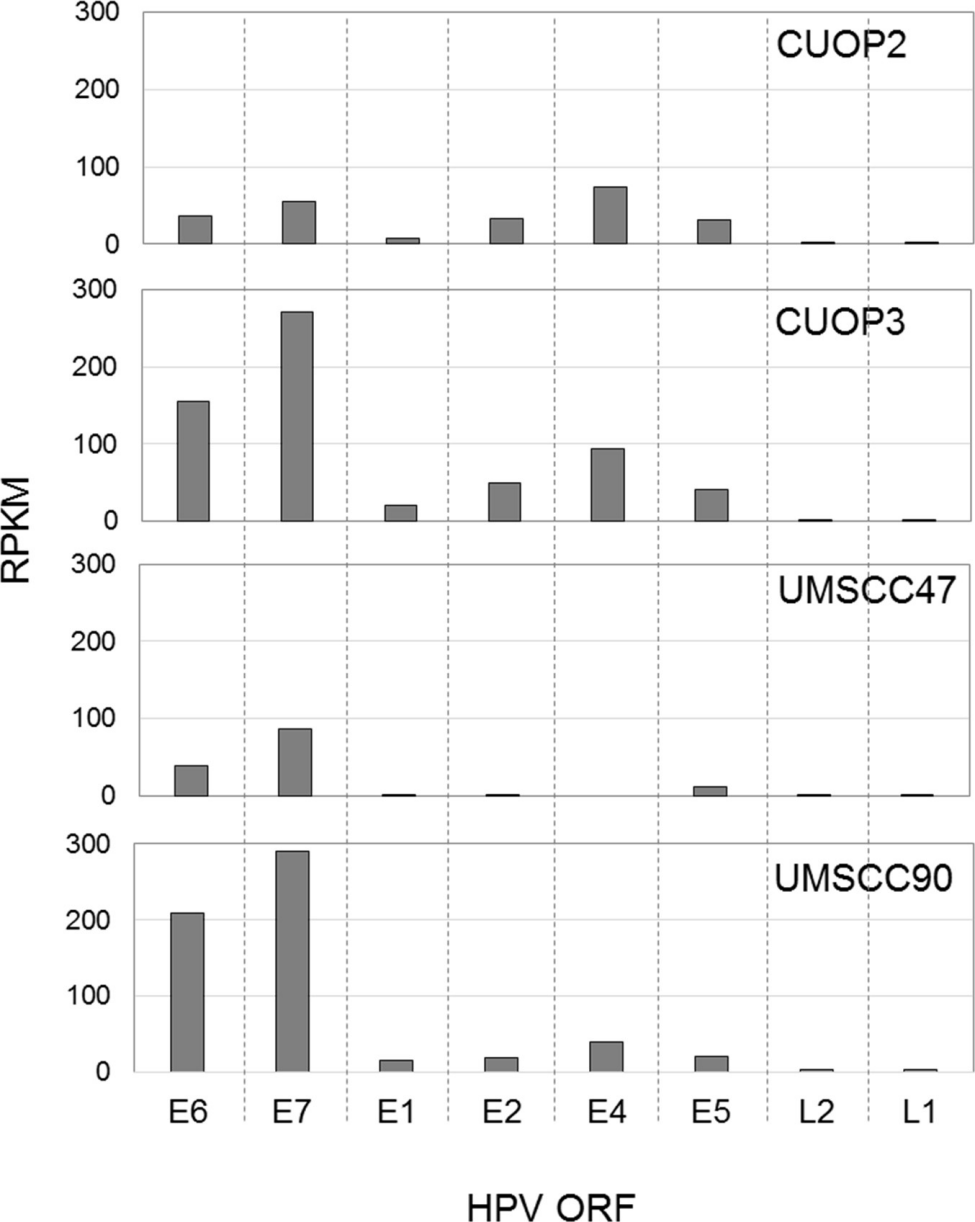
Quantification of HPV transcription. mRNA levels of HPV encoded genes were quantified in HPV-positive OPSCC cell lines. Reads Per Kilobase of transcript per million Mapped reads (RPKM) are plotted for the individual HPV open reading frames (ORF) of the four HPV-positive cell lines.

**Fig 2:**
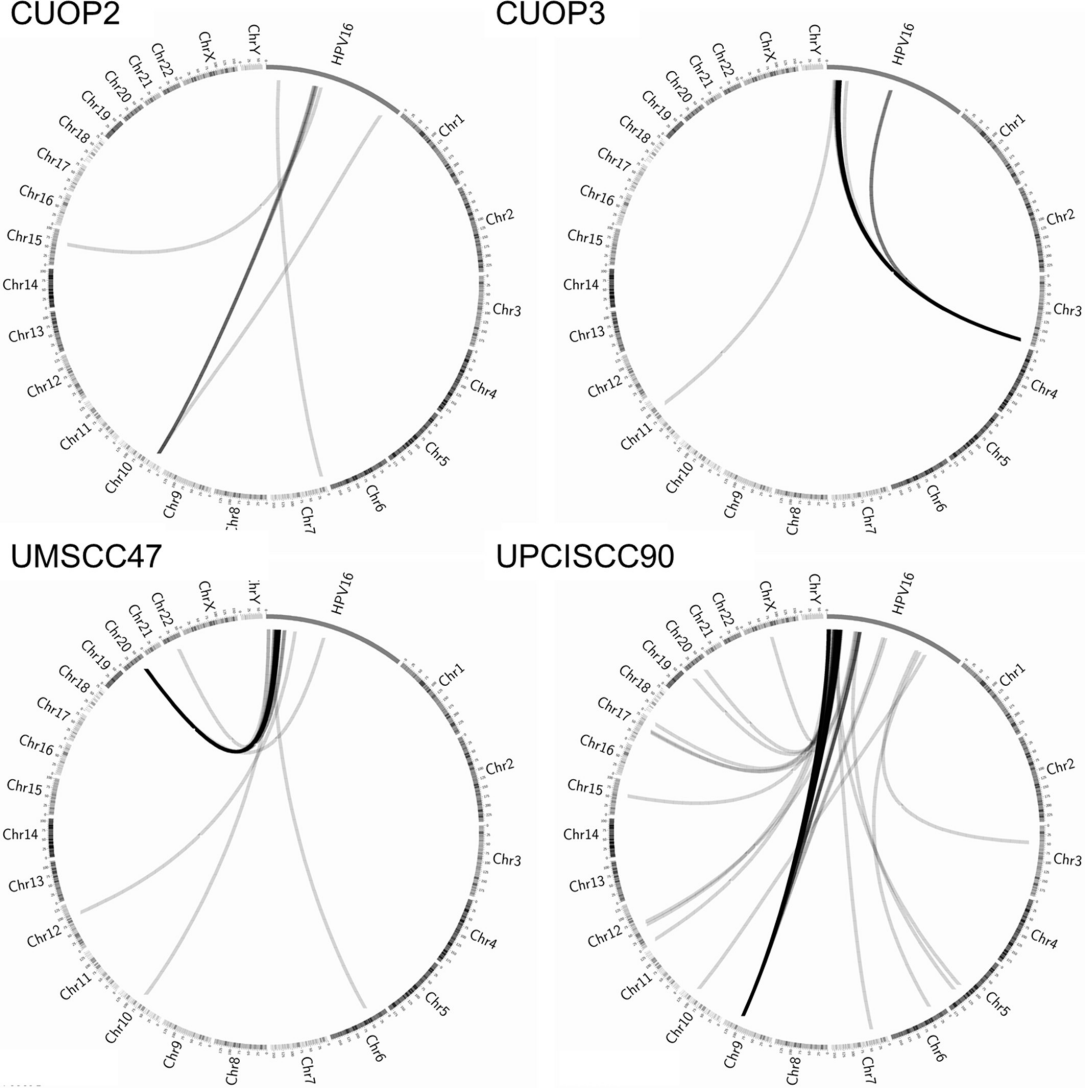
Determination of HPV integration state. Circos plots were used to illustrate fusion transcripts between HPV16 and human genome. HPV16 sequences and human chromosomes are not to scale. Fusion transcripts are represented as grey lines indicating where read ends mapped. Thicker/darker lines indicate multiple reads detected.

Paired-end mRNA sequencing was performed in our two novel HPV-positive cell-lines, CUOP2 and CUOP3, and in the two HPV-positive cell lines acquired from other laboratories, UMSCC47 and UPCISCC90, to assess the pattern of expression of HPV encoded genes (Fig 1), and determine whether fusion transcripts, arising from integrated viral DNA, were present (Fig 2). This analysis showed the highest levels of expression of HPV16 encoded oncogenes in the UMSCC90 and CUOP3 cell-lines. Fusion transcripts were identified in all four HPV-positive cell-lines indicating the presence of integrated virus (S1 Table), however the presence in several lines, especially CUOP2 and CUOP3, of transcripts encoded downstream of E6 and E7 suggests presence of full length HPV genomes either in integrated or episomal form.

**Fig 3:**
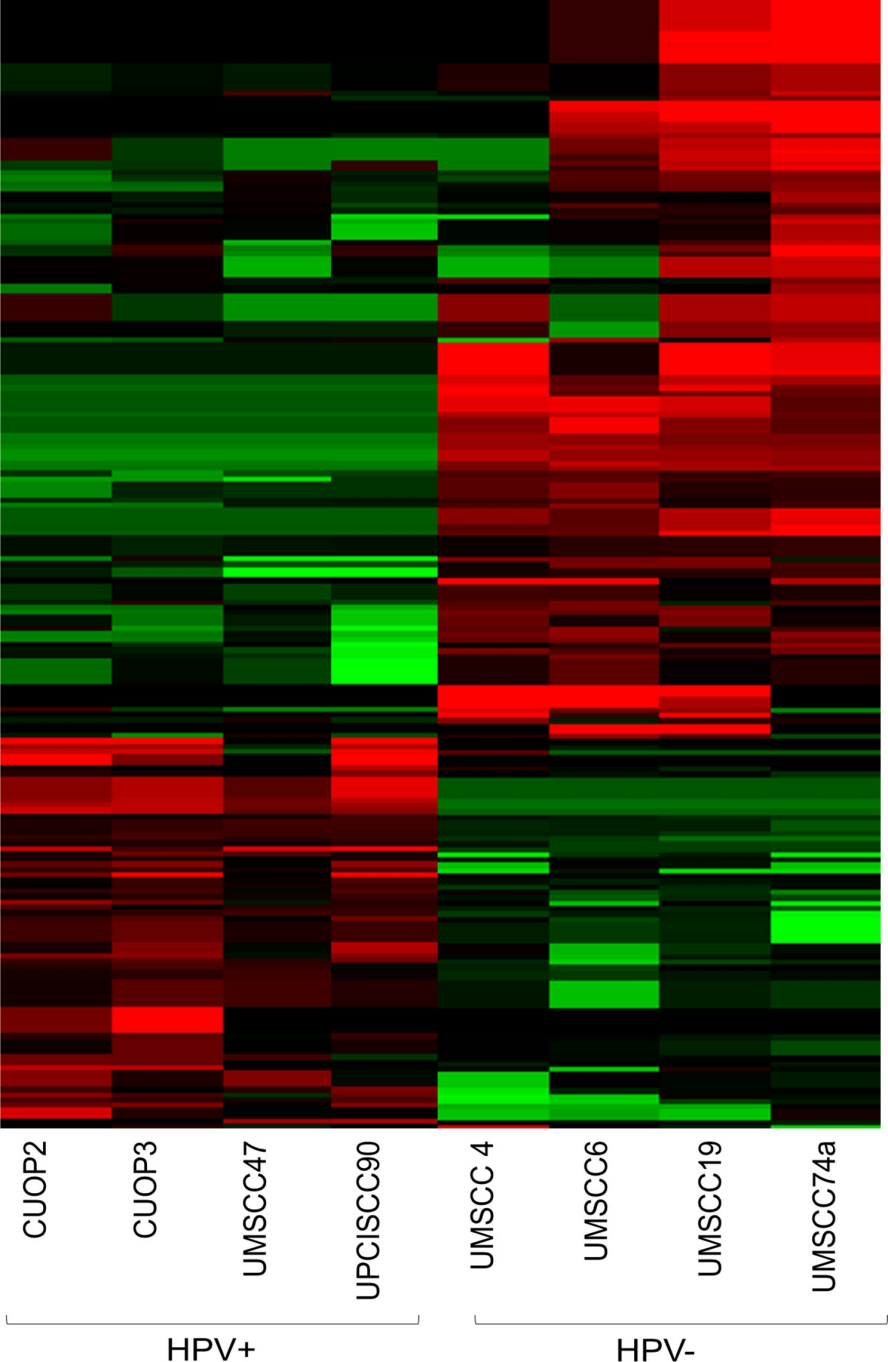
Gene expression in HPV positive and negative cell lines. The heatmap represents differential gene expression between HPV-positive and negative cell lines. Gene expression was normalised across the dataset: black represents the median, green represents expression below the median and red above median. After correction for multiple testing, 211 transcripts were significantly differentially expressed between the two groups (Benjamini–Hochberg FDR p<0.05).

mRNA-seq was also performed on the HPV-negative lines. The combined datasets were used to investigate differences in gene expression between HPV-positive and negative lines, and specifically to determine whether there were differences in expression of genes involved in repair of DNA DSB or SSB/base damage. After correction for multiple testing (Benjamini–Hochberg FDR p<0.05), 211 transcripts were significantly differentially expressed between HPV-positive and negative lines (Fig 3). These differences were investigated further using Gene Ontology Over Representation Analysis (GO-ORA). The differentially expressed genes were predominantly related to metabolic processes including glucoronidation, flavonoid and uronic acid metabolism. Several cellular processes including extracellular matrix (ECM) organisation and chromosome organisation were also significantly differentially regulated (see S2–S4 Tables for a full list of significant ontologies). Ontology directed heatmaps were generated for DSB repair, Base Excision Repair (BER), APOBEC activity, and p53-mediated signalling in response to DNA damage, but these analyses did not suggest any consistent and/or significant differences, between HPV-positive and negative cells (S2 Fig–S5 Fig).

**Fig 4:**
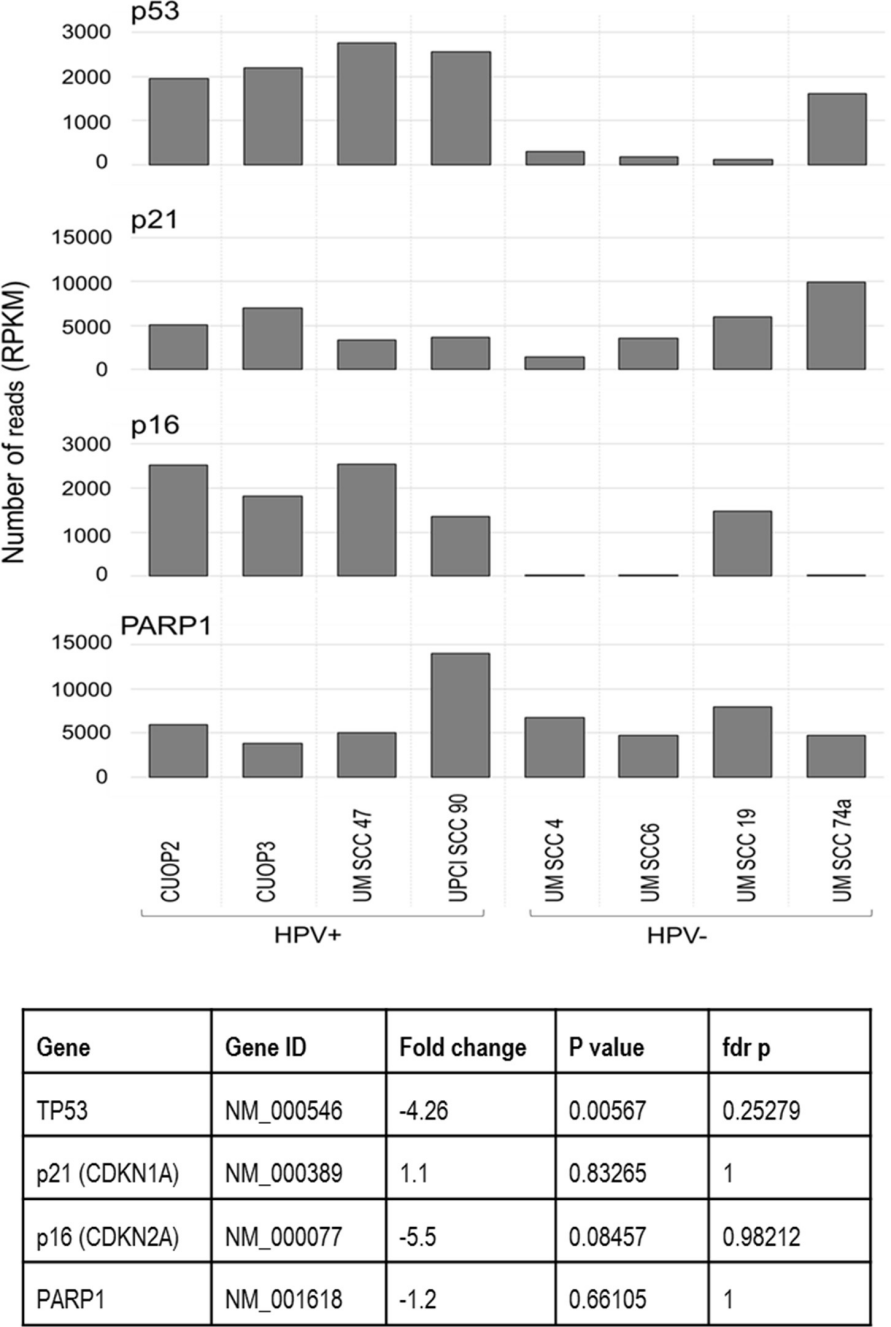
mRNA levels of p53, p21, p16 and PARP1 in HPV positive and negative cell lines. The number of reads, expressed as RPKM, for p53, p21, p16 and PARP1 was determined. Data are depicted as histograms with tabulated values showing fold change between the HPV-positive and negative groups, and significance by T-test with and without fdr adjustment for multiple tests.

**Fig 5:**
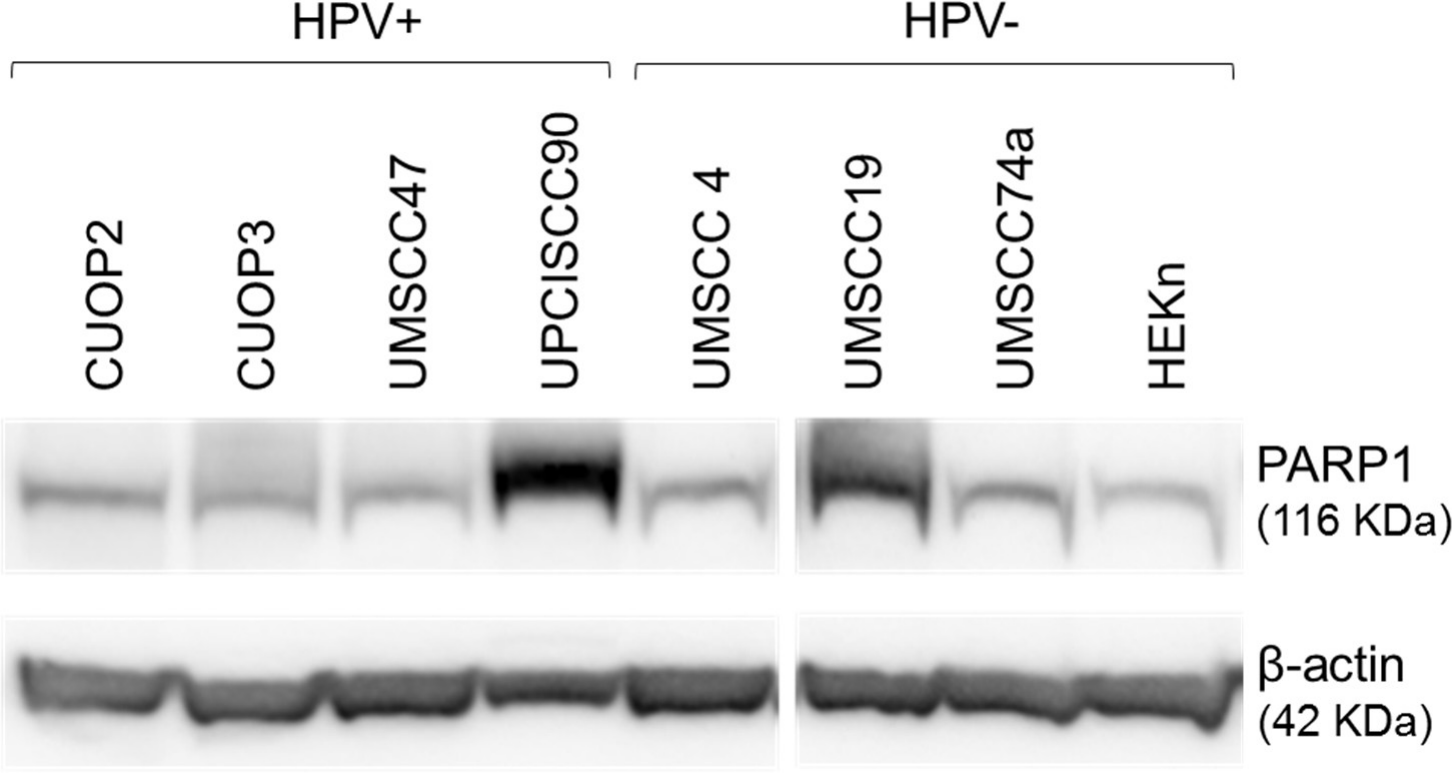
PARP1 protein in HPV-positive and negative cell lines. Protein was extracted from untreated cells and probed for PARP1 and β-actin (loading control). Image analysis was used to estimate relative levels of PARP1, and showed expression at >3-fold above the group median in UPCISCC90, and at >2-fold above the median in UMSCC19.

In consideration of their potential relevance to HPV status and DNA repair, transcript levels of several individual genes of interest were assessed (Fig 4). This demonstrated trends for greater abundance of transcripts for p53 and p16 in the HPV positive lines, but no differences in the levels of p21 or PARP1. In the HPV-positive UPCISCC90 cell-line, levels of PARP1 were 2–3 fold higher than in any other line, while the HPV-negative line UMSCC19 showed the second highest levels of PARP1 transcripts. Levels of PARP1 protein were also assessed by western blot, which also showed highest levels of PARP1 in UPCISCC90 and UMSCC19 (Fig 5).

### Response to Olaparib does not correlate with HPV status

**Fig 6:**
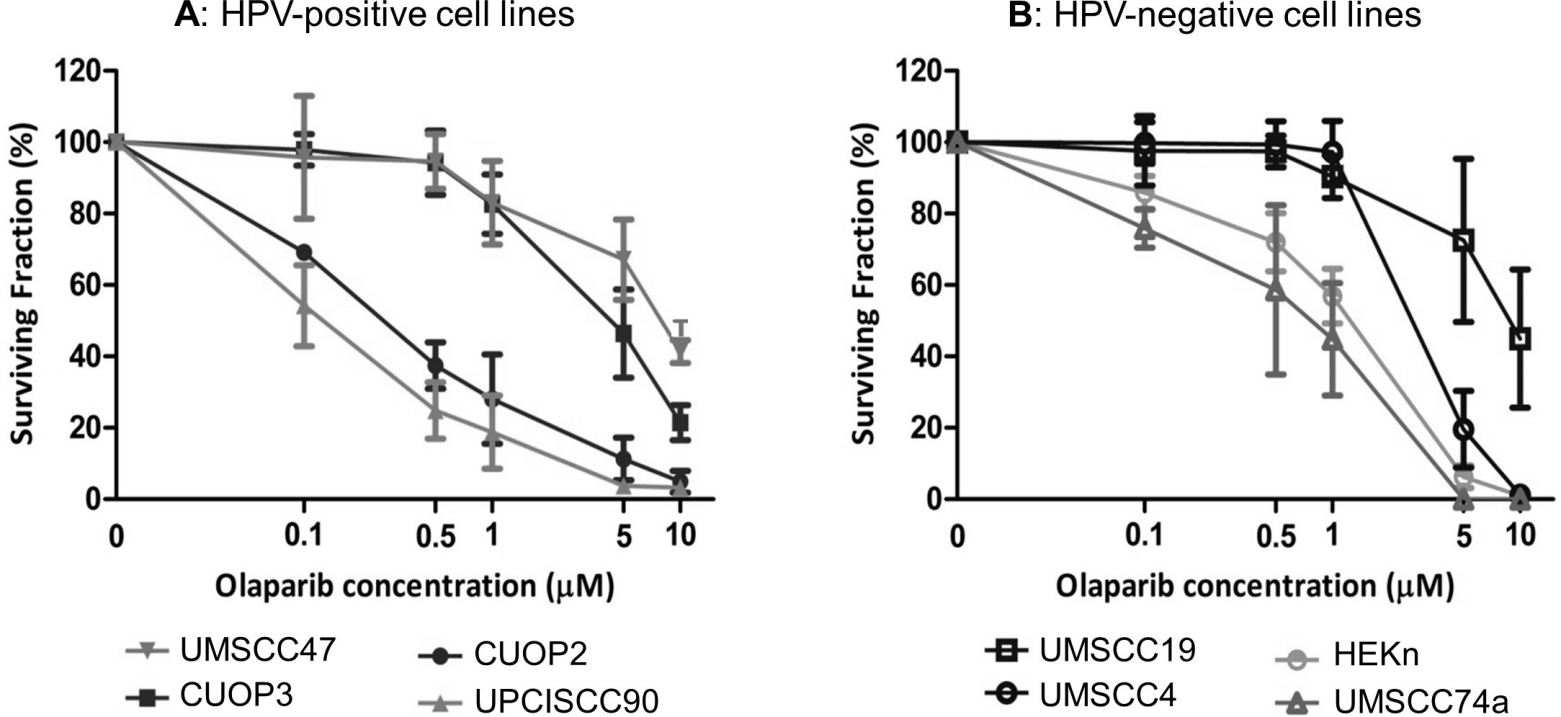
Surviving fractions of OPSCC cell lines in response to Olaparib treatment. Panel A: HPV-positive cells. At all doses between 0.1 & 10 μM Olaparib, PCOC2 and UPCISCC90 showed significantly lower surviving fractions compared to PCOC3 and UMSCC47 (p<0.05; two way ANOVA with Bonferroni post-test). Panel B: HPV-negative cells. At doses of 0.5 & 1 μM Olaparib, HEKn and UMSCC74a showed significantly lower surviving fractions compared to UMSCC19 and UMSCC4 (p<0.05; two way ANOVA with Bonferroni post-test). The results are representative of at least 3 experiments, all performed in triplicate. Mean and standard deviation are shown.

To determine whether sensitivity to the PARP inhibitor Olaparib correlated with HPV status, response to Olaparib was assessed at a range of concentrations (0.1–10 μM) using colony formation assays (Fig 6). All lines were sensitive to high doses of Olaparib (10 μM), which caused a reduction in colony formation of nearly 100% in 5 out of 8 lines. However, at doses between 0.5–1 μM, the surviving fractions differed significantly between lines (ANOVA p<0.001). In this dose range, three groups were apparent: a sensitive group defined as SF<40%, including two HPV-positive lines (UPCISCC90 and CUOP2), a medium sensitivity group with SF 40–80%, comprising an HPV-negative line and normal human epidermal keratinocytes (UMSCC74a and HEKn) and a resistant group with SF>80%, including 2 HPV-positive and 2 HPV-negative lines (UMSCC19, 4, 47 and CUOP3). This is only partially consistent with hypothesis that HPV status would correlate with response to Olaparib. It was also apparent that pre-treatment levels of PARP1 did not correlate with sensitivity to Olaparib, as the two lines with highest expression of PARP1, UPCISCC90 and UMSCC19, were in the sensitive and resistant groups respectively.

### Accumulation of DSB does not correlate with Olaparib sensitivity or HPV status

**Fig 7:**
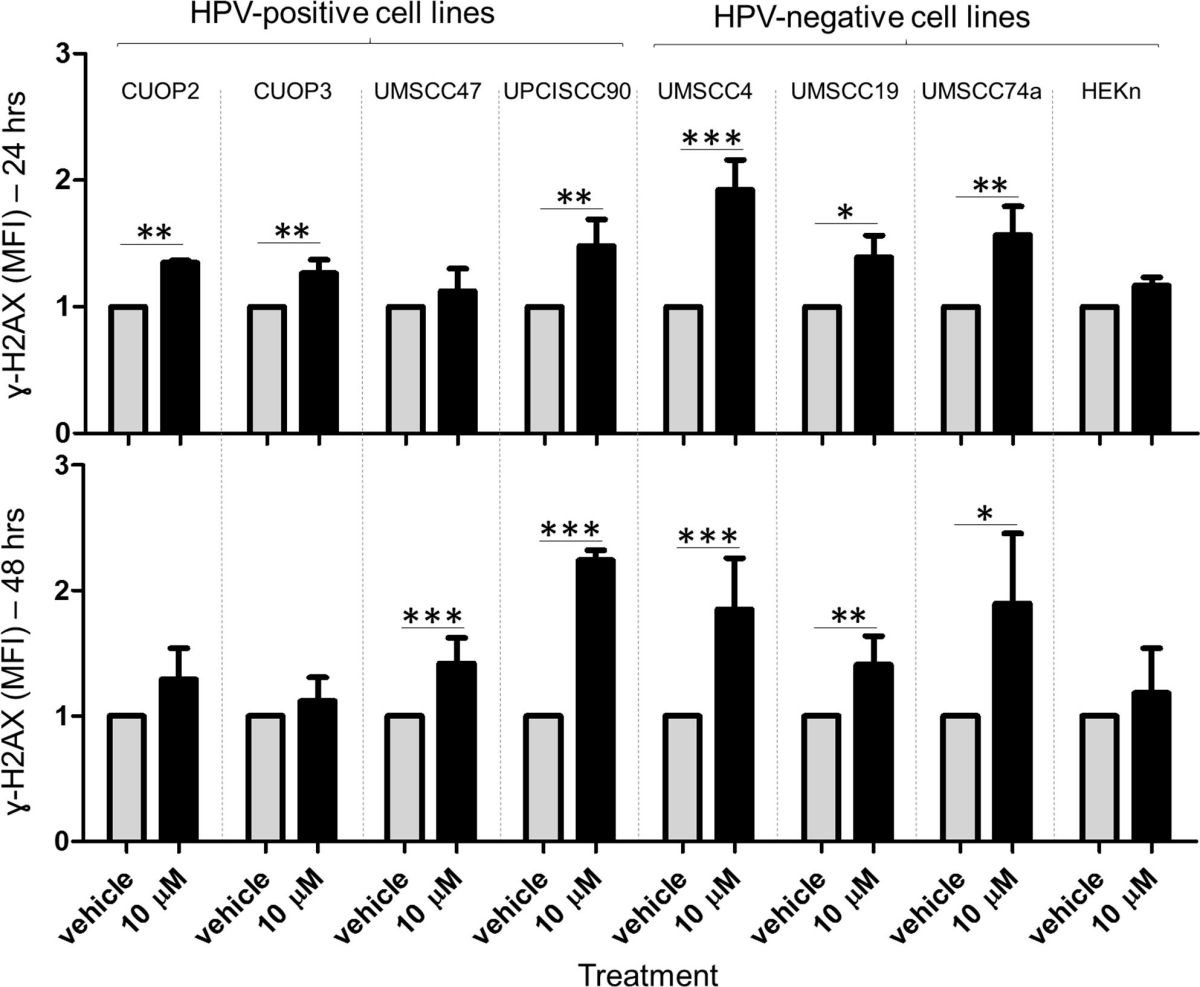
Induction of γ-H2AX following Olaparib treatment. Induction of γ-H2AX foci was assessed 24 & 48 hrs after treatment with 10 μM Olaparib. γ-H2AX levels were quantified using flow cytometry and are depicted as Mean Fluorescent Intensity (MFI) normalised to vehicle for each cell line. Data are representative of at least 3 independent experiments (ANOVA and Tukey post test, * P < 0.05, ** P<0.01, *** P<0.001). Error bars indicate standard deviation. Baseline levels of γ-H2AX in untreated cells are shown in S6 Fig.

Phosphorylation at the Ser-139 residue of the minor histone H2AX (termed γ-H2AX), signals and initiates repair of DSB, and is a key step in the DNA damage response. Detection of this phosphorylation event provides a specific and sensitive molecular marker for DNA damage, allowing assessment of initial damage and its subsequent resolution [28]. Levels of histone γ-H2AX were assessed at 24 and 48 hrs following Olaparib treatment (Fig 7). All OPSCC lines except UMSCC47 showed significantly increased levels of γ-H2AX 24 hrs following Olaparib treatment. In two of the HPV-positive lines, CUOP2 and 3, these levels returned to normal within 48 hrs, suggesting competent repair of DSB in these lines. For the two lines that were most sensitive to Olaparib, one showed competent repair (CUOP2) while the other (UPCISCC90) showed a marked inability to repair DSB; hence, sensitivity to Olaparib, did not appear to correlate with accumulation of DSB. Similarly, HPV-positive cells did not show impaired resolution of DSB relative to HPV-negative lines. In fact, the opposite trend was apparent; forty-eight hours after 10 μM Olaparib treatment, increased levels of γ-H2AX were evident in 2/4 HPV-positive lines and 3/3 HPV-negative OPSCC lines.

### Cell cycle arrest following Olaparib treatment partially correlates with sensitivity to Olaparib

**Fig 8:**
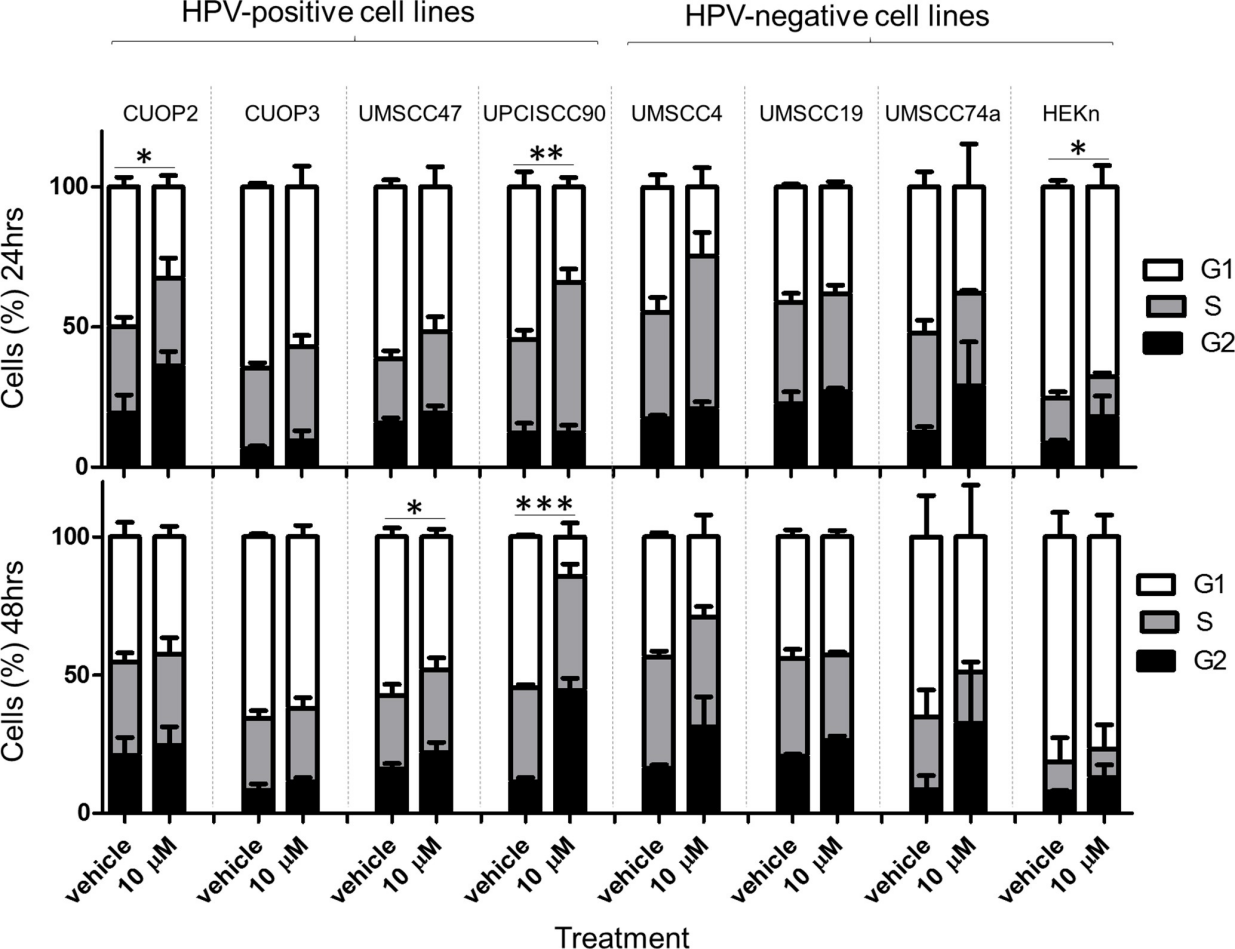
Cell cycle distribution following Olaparib treatment. The proportion of cells in G1, S and G2 for HPV-positive and negative cell lines 24 & 48 hrs post treatment with vehicle and 10 μM Olaparib are shown. Data are representative of at least 3 independent experiments (ANOVA and Tukey post test, * P < 0.05, ** P<0.01, *** P<0.001). Error bars indicate standard deviation.

The effects of Olaparib treatment on cell cycle distribution were assessed using flow cytometry (Fig 8). After 24 hrs, a correlation with sensitivity to Olaparib was apparent, with the two sensitive lines, CUOP2 and UPCISCC90, showing significant accumulation in G2 and S phase respectively. After 48 hrs, an increased G2 fraction was no longer evident in CUOP2, whereas UPCISCC90 displayed significant accumulation of cells in G2. For the cells that showed intermediate sensitivity to Olaparib, HEKn and UMSCC74a, HEKn showed a significant increase in cells in G2 at 24 hrs, and a non-significant increase at 48 hrs, while UMSCC74 showed non-significant increases in G2 fraction at both time-points. UMSCC47 showed significant expansion of the G2 fraction after 48 hrs. Overall, there seemed to be a general trend for accumulation of cells in G2 following Olaparib treatment, with the greatest effect visible in the most sensitive line (UPCISCC90).

### Accumulation of DSB and G2 arrest are not associated with apoptosis in HPV-positive cell-lines

**Fig 9:**
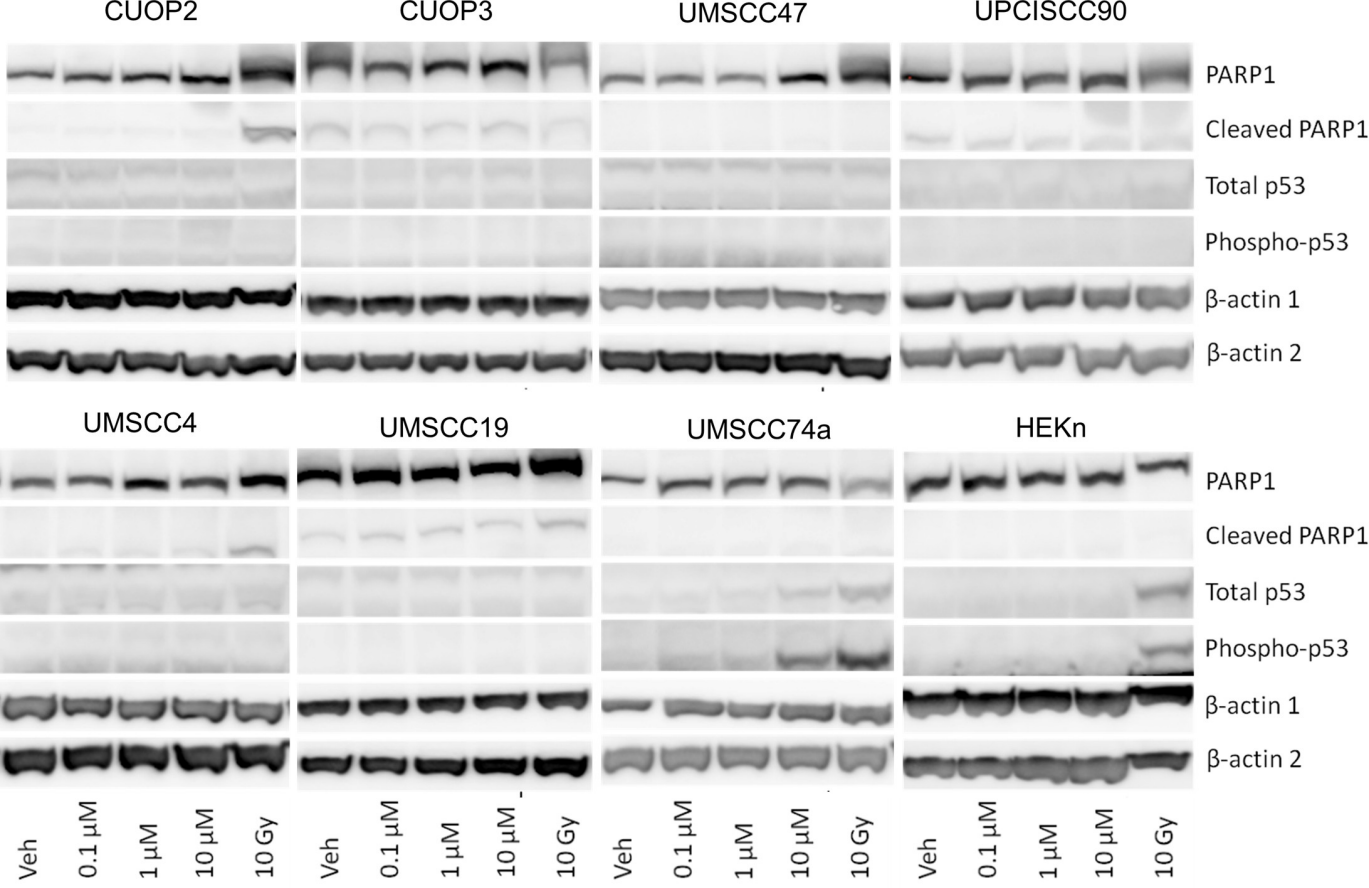
Protein levels of PARP1, cleaved PARP1, p53 and phospho-p53 48hrs following Olaparib treatment. Results for untreated and Olaparib dosed cells are shown. Cell lines treated with 10Gy ionising radiation, collected 24hrs post irradiation, were also included. Two membranes were used: one for PARP1 and cleaved PARP1 and β-actin 1; and the other for p53 and β-Actin 2.

It has been suggested that the superior response to therapy observed in patients with HPV-positive OPSCC may arise from an incomplete p53 phenotype arising from proteosomal degradation of p53, relative to the more complete phenotype conferred by mutation. According to this hypothesis residual low levels of p53 activity could promote an apoptotic response. To determine whether p53 mediated responses and apoptosis are relevant in the response to Olaparib, protein levels of p53, phosphorylated p53, and cleaved caspase were assessed (caspase mediated cleavage of PARP1 provides a sensitive and specific marker for apoptosis [29]) (Fig 9). Cells irradiated at a dose of 10 Gy were included to investigate whether a p53 mediated response could be detected in response to an alternative stimulus.

UMSCC74a was the only line that showed accumulation of p53 and phospho-p53, at 24 and 48 hrs, in response to 10 μM Olaparib (Fig 9), but this did not result in apoptosis, as assessed by cleavage of PARP1. Similarly, there was no microscopic evidence for apoptosis e.g. blebbing or cell shrinkage. p53 and phospho-p53 were also induced by Ionising Radiation (IR) in UMSCC74a cells. CUOP2 showed evidence of apoptosis, as indicated by cleaved PARP1, in response to IR, but not Olaparib. Conversely, HEKn cells displayed accumulation of p53 and phospho-p53 following IR, but no PARP1 cleavage indicating apoptosis. It was notable that even in UPCISCC90, substantial G2 arrest following 10 μM Olaparib was not associated with stabilisation and activation of p53 or apoptosis. Similarly, IR did not cause a detectable p53 or apoptotic response in this line. In summary, Olaparib treatment resulted in accumulation/activation of p53 in just one of the eight lines, and was associated with apoptosis in none of them.

## Discussion

Two novel cell-lines, CUOP2 and 3, were derived from HPV16-positive tonsil cancers from patients who are typical of the demographic increasingly affected by this disease i.e. both patients were relatively young, male, and with no or a low-level history of tobacco consumption. The molecular characteristics of the lines are also typical of HPV-positive tumours; both CUOP2 and 3 are p16-positive with wild-type p53. mRNA sequencing was used to demonstrate active transcription of the HPV16 E6 and E7 oncogenes. These lines were included in a panel of OPSCC cell-lines which was used to assess the effects of Olaparib treatment.

We had hypothesised that suboptimal repair of DSB in HPV-positive cells would confer sensitivity to PARP inhibition, and this would be associated with accumulation of DSB and G2 phase arrest. We also considered the possibility that an incomplete p53-negative phenotype would be associated with apoptosis following G2 arrest. We observed however, that sensitivity to Olaparib showed only partial correlation with HPV status: two of the four HPV-positive lines were classed as sensitive while two were classed as not sensitive to Olaparib.

There was no evidence to suggest less effective repair of DSB in HPV positive cells, in fact, the data seem to suggest the opposite. Three of the HPV-positive lines showed significantly increased levels of γ-H2AX 24 hrs after treatment with 10 μM Olaparib, but in two of these lines, levels had returned to baseline by 48 hrs, suggesting that the DSB had been repaired. In contrast, all of the HPV-negative OPSCC lines showed significantly increased levels of γ-H2AX at 24 hrs that were not repaired at 48 hrs. There was no evidence to support accumulation and activation of p53 in HPV-positive lines in response to Olaparib, or IR alone. Furthermore, the mRNA sequencing data did not indicate presence of functional p53 or any constitutive induction of DNA repair mechanisms in HPV-positive cells.

There is very little existing published *in vitro* data on the effects of PARP inhibition in OPSCC. In a study comparing response to 2–10 μM Veliparib in two HPV-positive vs one HPV-negative HNSCC cell-lines (UMSCC47, UPCISCC154 and UMSCC1 respectively), the HPV-negative line appeared approximately 1.5 fold less sensitive, and, contrary to our study, slower resolution of γ-H2AX foci in the HPV-positive cell-lines was observed [16]. A second study assessed the effects of Olaparib in four of the lines used in the current study (UMSCC 47, 6, 74a and UPCISCC90) but reported Olaparib, at doses up to 1 μM, as having no effect on cell proliferation, as determined using clonogenic assays, in any line, although this may be related to a shorter exposure to Olaparib (24 hrs versus the 7 days used in the current study) [15]. In a study by Wurster *et al*. the effects of 1 μM Olaparib were investigated in a panel of 10 HPV-negative HNSCC cell-lines [30]. These lines had been classified as homologous repair proficient or deficient, but none showed a substantial response to Olaparib, defined as >20% reduction in SF in clonogenic assays at 14 days. This suggests that DNA repair capacity is not predictive of response to Olaparib as monotherapy, but also that most HPV-negative HNSCC cell-lines may be resistant to Olaparib at dose levels at which some HPV-positive lines may be sensitive. It is hence possible that UMSCC74a may be unusual in showing “intermediate” sensitivity to Olaparib.

Whether p53 accumulates and causes apoptosis in HPV-positive HNSCC lines is controversial. One previous study reported accumulation of p53, caspase activation and increased annexin V binding in HPV-positive cells following 4 Gy IR [12]. However, a similar study reported no accumulation of p53 and the absence of caspase activation and PARP1 cleavage [11]. It should be noted however that the 15 HNSCC lines assessed represented a very diverse group that was not representative of typical HPV-positive oropharyngeal cancers, and only three lines were included in both studies. The relevance of these observations in the context of Olaparib monotherapy is uncertain, but accumulation of p53 and apoptosis were not provoked in response to the dosing regimen used in the current study.

UPCISCC90 and CUOP2 were sensitive to Olaparib at potentially therapeutic levels, however the mechanism for this effect appeared to differ between the two lines. In UPCISCC90 Olaparib sensitivity may be attributable to delayed resolution of DSB, as suggested by increased levels of γ-H2AX at 24 and 48 hrs, resulting in prolonged G2 arrest; alternatively, the high pre-treatment levels of PARP1 might indicate constitutive induction and reliance on the BER pathway. This is consistent with the observation that PARP is hyperactivated in cells with BRCA2 mutations and other defects in repair of DSB by homologous recombination [31]. APOBEC activity is induced in HPV-positive HNSCC [32], and this could result in upregulation of pathways contributing to repair of U:G mismatches, including BER [33]. However, this appears unlikely in the case of UPCISCC90, as there was no evidence of significant upregulation of APOBEC or BER pathway genes in the mRNA-seq data. The CUOP2 line showed competent repair of DSB with a transient G2 arrest, evident at 24 but not 48 hrs, and no evidence of apoptosis. The line also showed average levels of baseline PARP1. It is hence difficult to speculate as to the mechanism underlying the line’s highly significant sensitivity to Olaparib.

It is highly likely that the effects of HPV, in terms of response to therapy, will be dependent on the cell of origin [12]. This highlights the importance of using cellular models that best reflect the disease under investigation. This has been achieved in the current study by derivation of new cell-lines and use of exclusively oropharyngeal models. It is notable that due to the scarcity of treatment-naive OPSCC cell-lines, many previous studies have used HNSCC lines with diverse aetiologies and then drawn inferences regarding HPV-positive and negative OPSCC. We do however acknowledge that our study is based on only 8 cell-lines. There remains an urgent need for additional OPSCC cell-lines that represent the emerging demographic group.

Our findings suggest that some HPV-positive OPSCC cells may be sensitive to Olaparib at dose levels that may make monotherapy viable. This does not appear to be generally true of their HPV-negative counterparts. Only the results for UPCISCC90 were consistent with the original hypothesis, i.e. that in HPV-positive cells, treatment with Olaparib would cause accumulation of DSB, resulting in cell cycle arrest. For the other cell-lines, the data were more complex. The diverse responses across the cell-line panel suggests that caution should be exercised with regard to conclusions drawn in studies including only a small number of cell-lines. We did not observe any evidence to support an HPV-associated defect in repair of DSB, and our data are more consistent with the improved prognosis of HPV-positive OPSCC patients being associated with immune responses [34], than to defects in DNA repair or activation of p53-mediated apoptosis. This is also consistent with better patient outcomes being reported in HPV-positive patients who were treated surgically, as well as in those treated with chemo-radiotherapy [5,35–37]. Elucidation of the mechanisms responsible for sensitivity to Olaparib remains important, as this may allow identification of predictive markers to allow targeting of this treatment to patients in whom it will be effective. Without a predictive marker, our preclinical data suggest that monotherapy with Olaparib would not be effective in the majority of patients.

## Supporting information

S1 Fig CUOP2 and CUOP3 p16 immunohistochemistry. (DOCX)

S2 Fig Differential gene expression in the DSB repair pathway between HPV-positive and negative cells. (DOCX)

S3 Fig Differential gene expression in the BER pathway between HPV-positive and negative cells. (DOCX)

S4 Fig Differential gene expression in the APOBEC pathway between HPV-positive and negative cells. (DOCX)

S5 Fig Differential gene expression in p53-mediated signalling in response to DNA damage between HPV-positive and negative cells. (DOCX)

S6 Fig Basal level of γ-H2AX in OPSCC cell lines. (DOCX)

S1 Table HPV integration sites in UPCISCC90, CUOP2, CUOP3 and UMSCC47. (DOCX)

S2 Table Significant biological process ontologies (HPV-positive vs negative cell-lines). (DOCX)

S3 Table Significant molecular function ontologies (HPV-positive vs negative cell-lines). (DOCX)

S4 Table Significant cellular component ontologies (HPV-positive vs negative cell-lines). (DOCX)
