# Supplementary material for: Sensitivity to inhibition of DNA repair by Olaparib in novel oropharyngeal cancer cell lines infected with Human Papillomavirus
Source: PLoS One. 2018 Dec 13;13(12):e0207934. doi: 10.1371/journal.pone.0207934 (PMC6292594; doi:10.1371/journal.pone.0207934)
Supplement: S1 Fig — (DOCX) [file pone.0207934.s001.docx]

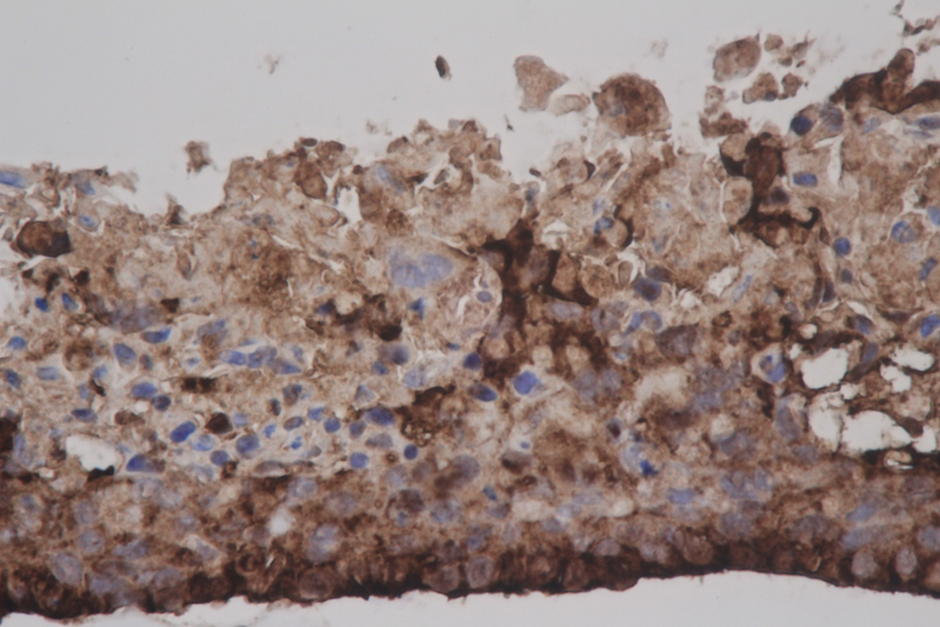


**A**


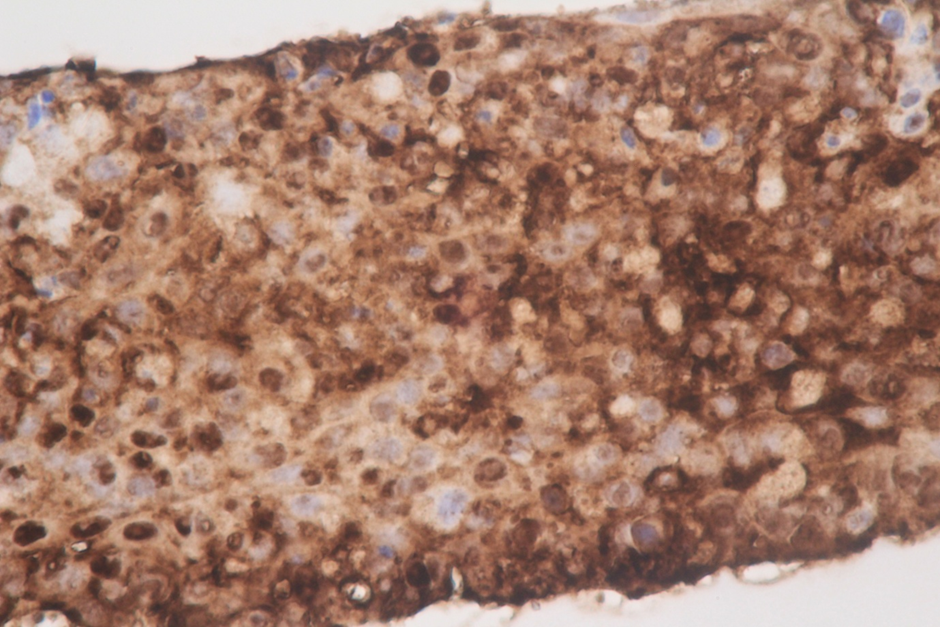


**B**

**S1 Figure.**

**CUOP2 and CUOP3 p16 immunohistochemistry**

*P16 immunohistochemistry was performed on cells cultured as organotypic rafts. Staining was performed by UCL Advanced Diagnostics according to standard protocols and with appropriate controls. Slides were scored by an experience histopathologist according to standard published criteria (>70% diffuse staining).*

*Panel A: CUOP2*

*Panel B: CUOP3*
