# Supplementary material for: Sensitivity to inhibition of DNA repair by Olaparib in novel oropharyngeal cancer cell lines infected with Human Papillomavirus
Source: PLoS One. 2018 Dec 13;13(12):e0207934. doi: 10.1371/journal.pone.0207934 (PMC6292594; doi:10.1371/journal.pone.0207934)
Supplement: S2 Fig — (DOCX) [file pone.0207934.s002.docx]

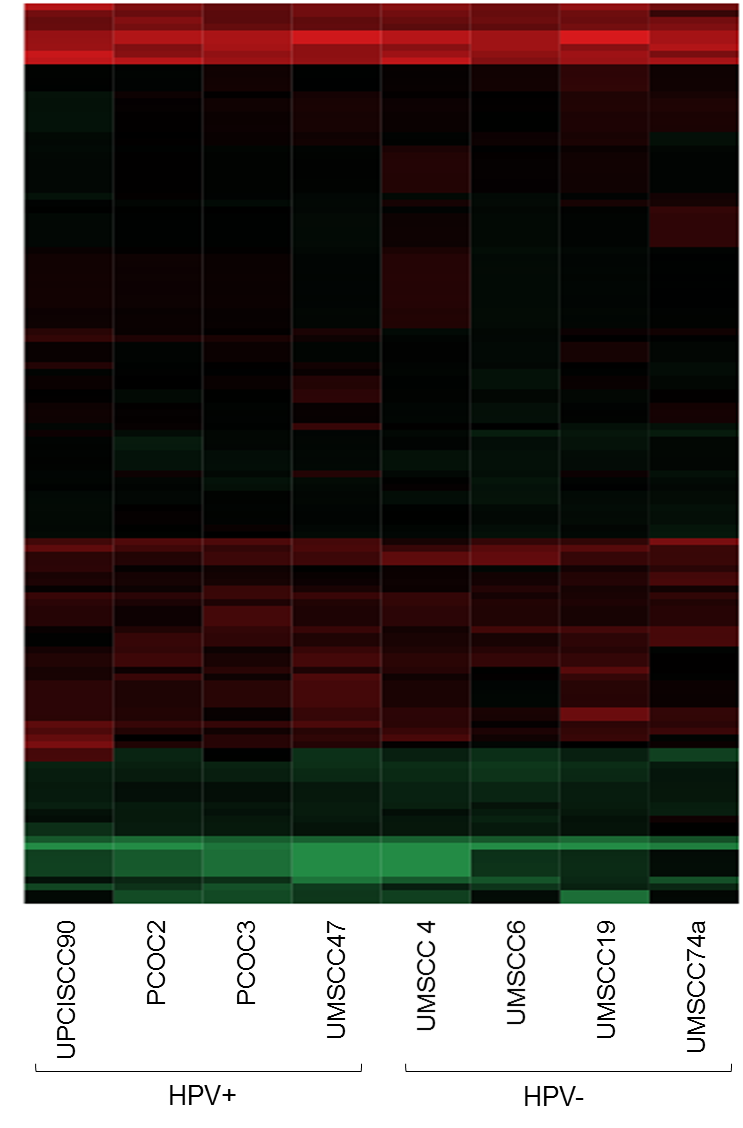


**S2 Figure.**

**Differential gene expression in the DSB repair pathway between HPV-positive and negative cells**

*The heatmap represents expression of the 56 genes in the ontology “Double strand break repair – GO:0006302” between HPV-positive and negative cell lines. Gene expression was normalised across the dataset: black represents the median, green represents expression below the median and red above median. After correction for multiple testing, 0 transcripts were significantly differentially expressed between the two groups (FDR p<0.05).*
