# Supplementary material for: Sensitivity to inhibition of DNA repair by Olaparib in novel oropharyngeal cancer cell lines infected with Human Papillomavirus
Source: PLoS One. 2018 Dec 13;13(12):e0207934. doi: 10.1371/journal.pone.0207934 (PMC6292594; doi:10.1371/journal.pone.0207934)
Supplement: S6 Fig — (DOCX) [file pone.0207934.s006.docx]

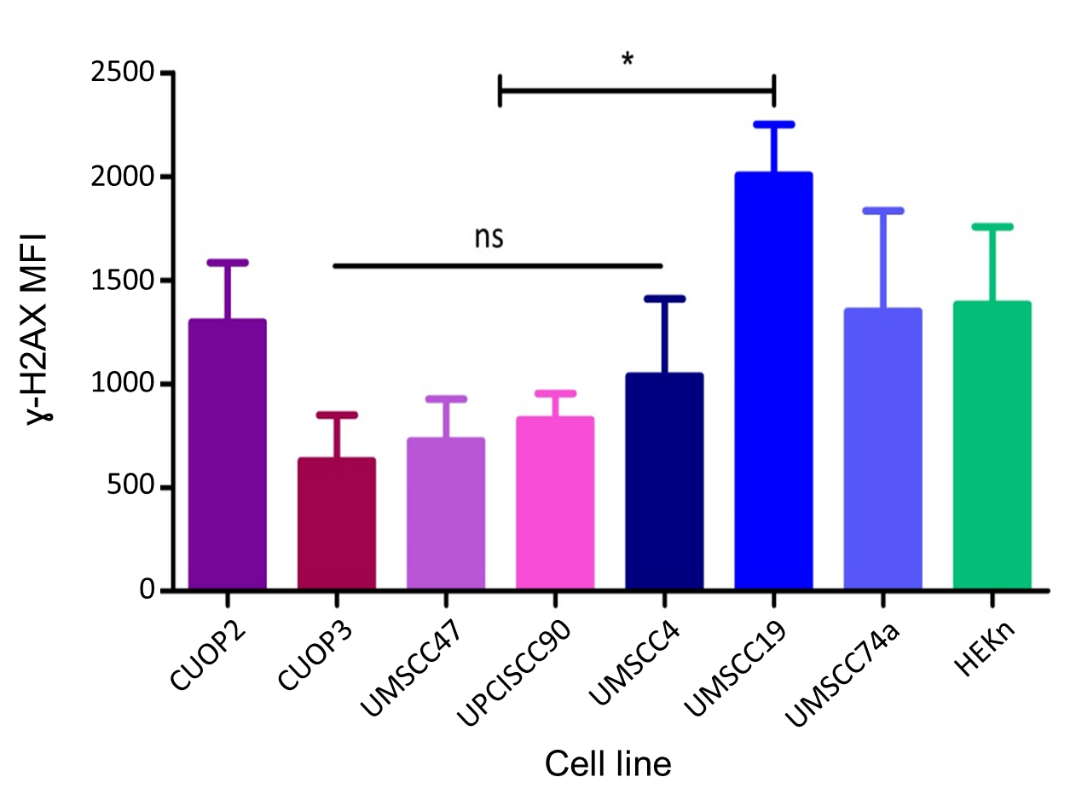


**S6 Figure.**

**Basal level of** *γ***-H2AX in OPSCC cell lines**

*UMSCC19 showed a significantly higher level of γ-H2AX than CUOP3, UMSCC47, UPCISCC90 and UMSCC4 (*indicated in figure: p<0.05, ANOVA with Bonferroni’s post-test). PCOC2, UMSCC74a and the normal keratinocyte line HEKn showed intermediate levels of γ-H2AX. Results are representative of at least 3 experiments. Error bars denote standard deviation. MFI indicates Mean Fluorescent Intensity.*
