# Supplementary material for: Sensitivity to inhibition of DNA repair by Olaparib in novel oropharyngeal cancer cell lines infected with Human Papillomavirus
Source: PLoS One. 2018 Dec 13;13(12):e0207934. doi: 10.1371/journal.pone.0207934 (PMC6292594; doi:10.1371/journal.pone.0207934)
Supplement: S1 Table — (DOCX) [file pone.0207934.s007.docx]

**S1 Table****.**

**HPV integration sites in UPCISCC90, CUOP2, CUOP3 and UMSCC47**

| **Cell-line** | **HPV gene** | **Human chromosome** | **Human gene^1^** |
| --- | --- | --- | --- |
| UPCISCC90 | E1 | 9 | C9orf156 Exon3 |
|  |  | 9 | C9orf156 Intron2 |
|  | E6 | 9 | C9orf156 Exon3 |
|  | E7 | 9 | C9orf156 Exon3 |
|  |  | 9 | Non coding +- 200bp before FOXE1 |
| CUOP2 | E2 | 10 | YMEL1 Intron 6 and 7 |
| CUOP3 | E7 | 20 | CEPBP |
| UMSCC47 | E7 | 3 | TP63 Exon 11 |

^1^ Insertion events with at least 5 mapped reads
