# Supplementary material for: Sensitivity to inhibition of DNA repair by Olaparib in novel oropharyngeal cancer cell lines infected with Human Papillomavirus
Source: PLoS One. 2018 Dec 13;13(12):e0207934. doi: 10.1371/journal.pone.0207934 (PMC6292594; doi:10.1371/journal.pone.0207934)
Supplement: S2 Table — (DOCX) [file pone.0207934.s008.docx]

S2 Table.

Significant Biological Process ontologies (HPV-positive vs negative cell-lines)

| **GO ID** | **Term** | **p.fdr ^1^** |
| --- | --- | --- |
| GO:2001029 | regulation of cellular glucuronidation | 5.07E-11 |
| GO:0052696 | flavonoid glucuronidation | 1.22E-08 |
| GO:0052695 | cellular glucuronidation | 1.32E-07 |
| GO:0009812 | flavonoid metabolic process | 1.94E-07 |
| GO:0006063 | uronic acid metabolic process | 5.03E-07 |
| GO:0019585 | glucuronate metabolic process | 5.03E-07 |
| GO:0030198 | extracellular matrix organization | 1.34E-06 |
| GO:0043062 | extracellular structure organization | 1.34E-06 |
| GO:0045922 | negative regulation of fatty acid metabolic process | 3.86E-06 |
| GO:0051552 | flavone metabolic process | 4.56E-06 |
| GO:0009813 | flavonoid biosynthetic process | 4.56E-06 |
| GO:0045912 | negative regulation of carbohydrate metabolic process | 4.72E-06 |
| GO:0042573 | retinoic acid metabolic process | 1.22E-05 |
| GO:0010677 | negative regulation of cellular carbohydrate metabolic process | 1.83E-05 |
| GO:0045833 | negative regulation of lipid metabolic process | 1.05E-04 |
| GO:0006805 | xenobiotic metabolic process | 2.67E-04 |
| GO:0071466 | cellular response to xenobiotic stimulus | 3.36E-04 |
| GO:0009410 | response to xenobiotic stimulus | 4.34E-04 |
| GO:0009888 | tissue development | 9.29E-04 |
| GO:0032787 | monocarboxylic acid metabolic process | 1.86E-03 |
| GO:0006631 | fatty acid metabolic process | 1.86E-03 |
| GO:0019217 | regulation of fatty acid metabolic process | 3.26E-03 |
| GO:0022617 | extracellular matrix disassembly | 3.53E-03 |
| GO:0001523 | retinoid metabolic process | 1.59E-02 |
| GO:0045143 | homologous chromosome segregation | 1.94E-02 |
| GO:0006109 | regulation of carbohydrate metabolic process | 1.94E-02 |
| GO:0016101 | diterpenoid metabolic process | 2.58E-02 |
| GO:0010511 | regulation of phosphatidylinositol biosynthetic process | 2.83E-02 |
| GO:0070192 | chromosome organization involved in meiotic cell cycle | 3.03E-02 |
| GO:0043436 | oxoacid metabolic process | 3.79E-02 |
| GO:0034754 | cellular hormone metabolic process | 4.31E-02 |
| GO:0006082 | organic acid metabolic process | 4.54E-02 |
| GO:0010675 | regulation of cellular carbohydrate metabolic process | 4.54E-02 |
| GO:0006721 | terpenoid metabolic process | 4.54E-02 |
| GO:0019752 | carboxylic acid metabolic process | 4.54E-02 |
| GO:0007129 | synapsis | 4.54E-02 |
| GO:0007126 | meiotic nuclear division | 4.54E-02 |

^1^The p value represents data corrected for multiple testing and false discovery. 37 GO were significantly different (p.fdr <0.05) between the two groups.
