# Supplementary material for: Sensitivity to inhibition of DNA repair by Olaparib in novel oropharyngeal cancer cell lines infected with Human Papillomavirus
Source: PLoS One. 2018 Dec 13;13(12):e0207934. doi: 10.1371/journal.pone.0207934 (PMC6292594; doi:10.1371/journal.pone.0207934)
Supplement: S3 Table — (DOCX) [file pone.0207934.s009.docx]

S3 Table.

Significant Molecular Function ontologies (HPV-positive vs negative cell-lines)

| **GO ID** | **Term** | **p.fdr^1^ value** |
| --- | --- | --- |
| GO:0015020 | glucuronosyltransferase activity | 1.11E-06 |
| GO:0005501 | retinoid binding | 1.06E-04 |
| GO:0019840 | isoprenoid binding | 1.67E-04 |
| GO:0001972 | retinoic acid binding | 1.41E-03 |
| GO:0008194 | UDP-glycosyltransferase activity | 1.02E-02 |

^1^The p value represents data corrected for multiple testing and false discovery. Five GO were significantly different (p.fdr <0.05) between the two groups.
