# Supplementary material for: Sensitivity to inhibition of DNA repair by Olaparib in novel oropharyngeal cancer cell lines infected with Human Papillomavirus
Source: PLoS One. 2018 Dec 13;13(12):e0207934. doi: 10.1371/journal.pone.0207934 (PMC6292594; doi:10.1371/journal.pone.0207934)
Supplement: S4 Table — (DOCX) [file pone.0207934.s010.docx]

S4 Table.

Significant Cellular Component ontologies (HPV-positive vs negative cell-lines)

| GO ID | **Term** | **p.fdr^1^ value** |
| --- | --- | --- |
| GO:0005578 | proteinaceous extracellular matrix | 1.86E-03 |
| GO:0031012 | extracellular matrix | 3.53E-03 |
| GO:0044421 | extracellular region part | 1.32E-02 |
| GO:0005576 | extracellular region | 1.72E-02 |
| GO:0000795 | synaptonemal complex | 4.54E-02 |

^1^The p value represents data corrected for multiple testing and false discovery. Five GO were significantly different (p.fdr <0.05) between the two groups.
